# Anti-Human Endoglin (hCD105) Immunotoxin—Containing Recombinant Single Chain Ribosome-Inactivating Protein Musarmin 1

**DOI:** 10.3390/toxins8060184

**Published:** 2016-06-10

**Authors:** Begoña Barriuso, Pilar Antolín, F. Javier Arias, Alessandra Girotti, Pilar Jiménez, Manuel Cordoba-Diaz, Damián Cordoba-Diaz, Tomás Girbés

**Affiliations:** 1 Department of Biochemistry and Molecular Biology, University of Valladolid, 47005 Valladolid, Spain; barmagma@jcyl.es (B.B.); mantolin@umh.es (P.A.); arias@bioforge.uva.es (F.J.A.); 2 Bioforge, University of Valladolid, Spain and Networking Research Center on Bioengineering, Biomaterials and Nanomedicine (CIBER-BBN), 47002 Valladolid, Spain; agirotti@bioforge.uva.es; 3 Department of Nutrition and Bromatology, University of Valladolid, 47005 Valladolid, Spain; pilarj@bio.uva.es; 4 Department of Pharmacy & Pharmaceutical Technology and University Institute of Industrial Pharmacy (IUFI), Complutense University of Madrid, 28040 Madrid, Spain; mcordoba@farm.ucm.es (M.C.-D.); damianco@farm.ucm.es (D.C.-D.)

**Keywords:** recombinant musarmin, endoglin, CD105, 44G4, anti-endoglin monoclonal antibody, anti-tumor therapy, cancer therapy

## Abstract

Endoglin (CD105) is an accessory component of the TGF-β receptor complex, which is expressed in a number of tissues and over-expressed in the endothelial cells of tumor neovasculature. Targeting endoglin with immunotoxins containing type 2 ribosome-inactivating proteins has proved an effective tool to reduce blood supply to B16 mice tumor xenografts. We prepared anti-endoglin immunotoxin (IT)—containing recombinant musarmin 1 (single chain ribosome-inactivating proteins) linked to the mouse anti-human CD105 44G4 mouse monoclonal antibody via *N*-succinimidyl 3-(2-pyridyldithio) propionate (SPDP). The immunotoxin specifically killed L929 fibroblast mouse cells transfected with the short form of human endoglin with IC_50_ values in the range of 5 × 10^−10^ to 10^−9^ M.

## 1. Introduction

Attacking cancer cells with drugs can be performed either directly by targeting cells or indirectly by destroying the new blood vessels induced by the tumor which each support hundreds of cancer cells. The latter strategy is known as the antiangiogenic approach to cancer therapy [1]. This may be performed using cytotoxic agents such as antibodies aimed at the vasculature targets and immunotoxins (ITs). ITs are formed by at least two parts, one a carrier, such as an antibody or a ligand able to recognize and bind specific targets, and the other a toxic moiety, such as plant or bacterial toxins, which are able to kill target cells [2,3,4,5]. In conventional ITs, both parts are linked by chemical means, while in recombinant ITs, the two parts are fused by genetic engineering. In conventional ITs, a number of ribosome-inactivating proteins (RIPs) have been used as a toxic moiety [5]. In recombinant ITs, bacterial toxins such as those of *Pseudomonas exotoxin* or from diphtheria toxin and plant ribosome-inactivating proteins such as gelonin have been used [6,7,8].

The TGF-β co-receptor endoglin (CD105) is expressed in several mammalian tissues related to the proliferation of new vasculature [9]. Endoglin is considered a marker for evaluating microvessel density in tumors [10], and has also been reported as a target of tumor neovasculature for cancer therapy [11,12,13,14]. Recent research has supported the idea that silencing it promotes a significant antitumor effect on murine mammary adenocarcinoma [15].

Targeting endoglin over-expressed in tumor neovasculature has been used for therapeutic purposes with anti-endoglin antibodies [16], endoglin-targeted radioimmunotherapy [17], and anti-endoglin antibody–containing ITs [18]. Efficient conventional anti-endoglin ITs containing ricin A-chain [19] and the type 2 RIPs nigrin b [12,20] and ebulin l [21] have been constructed. In the present work, we report proof of the validity of the new IT (rMU1-44G4), containing rMU1 as a toxic moiety linked to an anti-human CD105 monoclonal antibody (44G4), on cultured mouse L929 fibroblasts transfected with the short form of CD105 [22]. rMU1, a recombinant form expressed in *Escherichia coli,* of musarmin 1 (MU1) present in bulbs of the plant *Muscari armeniacum* L. Miller was able to retain full activity when tested on mammalian ribosomes both in translation and in N-glycosidase activities [23].

## 2. Results and Discussion

**Figure 1:**
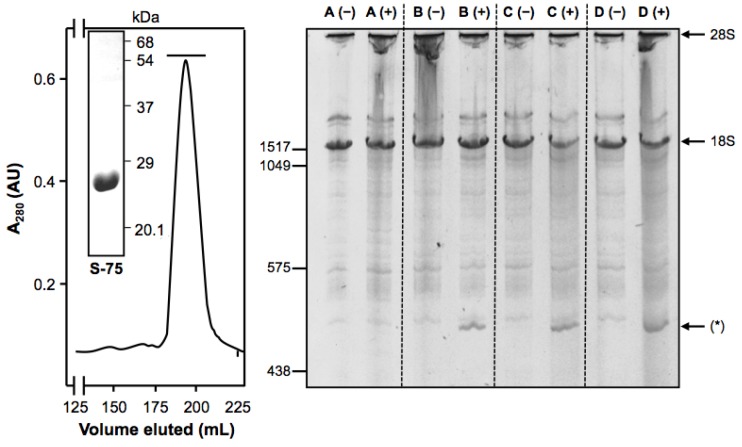
Molecular characterization of rMU1. **Left panel**: Superdex 75 chromatography of rMU1 and SDS-PAGE analysis of the preparation (inset); the horizontal bar indicates the collected factions. **Right panel**: *N*-glycosidase of rMU1 on rabbit reticulocyte ribosomes; A: control; B: soluble rMU1; C: rMU1 from solubilized inclusion bodies; D: Saporin-S5; (+) indicates the treatment of the lysates with acid aniline and (*) indicates the Endo fragment; numbers on the left indicate base pairs and on the right the rRNA type.

Preparation of the rMU1 from the cytosolic insoluble fraction (inclusion bodies), upon extraction, solubilization and refolding, yielded 20 mg/L of rMU1 [23]. As shown in Figure 1 (left), the rMU1 batch used to construct immunotoxins was homogeneous as judged by gel filtration through Superdex 75, giving a symmetric peak, and SDS-PAGE, with a single band of apparent Mr of 28,000 Da. As also shown in Figure 1 (right), rMU1 promotes the release of the Endo rRNA fragment, which is diagnostic of RIP *N*-glycosidase action on the 28S rRNA. For comparison purposes, we also include type 1 RIP Saporin S5 [24] in the experiment.

The first step in immunotoxin construction was to determine the number of sulfide residues introduced by the linker into the recombinant RIP and their effect on RIP activity. The study was conducted with rMU1 and *N*-succinimidyl 3-(2-pyridyldithio)propionate (SPDP) as the linker. It was found that 1–1.5–SH residues per molecule of RIP was the best situation (data not shown). With that ratio, the IC_50_ of rMU1 on rabbit reticulocyte lysates was 30 nM.

**Figure 2:**
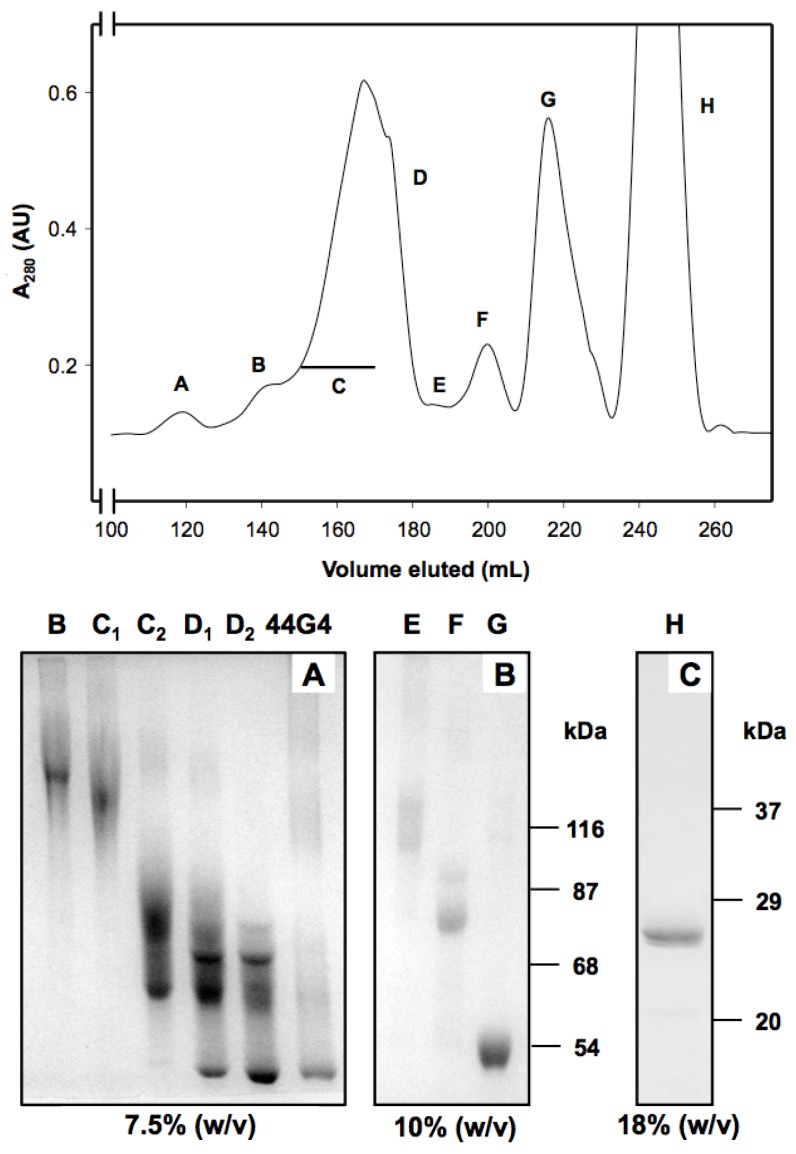
Purification (**upper panel**) and SDS-PAGE analysis (**lower panel**) of the rMU1-44G4 immunotoxin. **Upper panel**: The reaction mixtures were carried out as indicated in Materials and Methods and purified by gel filtration chromatography on Superdex 200 HiLoad. Letters in the chromatogram of the upper panel indicate peaks analyzed by SDS-PAGE; the fractions collected in each peak were: B (high molecular weight aggregates of 44G4 and RMU1), 135–140 mL; C1 (medium molecular weight conjugates rMU1-44G4), 150–155 mL; C2, (low molecular weight conjugates rMU1-44G4), 165–170 mL; D1 (low molecular weight conjugates rMU1-44G4), 170–175 mL; D2 (low molecular weight conjugates rMU1-44G4), 175–180 mL; E (rMU1 tetramer), 185–190 mL; F (rMU1 trimer), 192–207 mL; G (rMU1 dimer), 207–230 mL; H (rMU1), 235–260 mL. Both C1 and C2 were mixed and used as immunotoxin; **Lower panel**: Proteins contained in the indicated peaks were analyzed by SDS-PAGE using three different concentrations of acrylamide: 7.5% (*w*/*v*) (**A**); 10% (*w*/*v*) (**B**); 18% (*w*/*v*) (**C**); numbers on the right indicate the apparent Mr; numbers in parentheses indicate the estimated size. The amount of protein loaded into each well was: 30 micrograms in lanes B, C1, C2, D1, D2; four micrograms in lane H; 10 micrograms in lanes G, F and E. Numbers on the right indicate the corresponding apparent Mr values of the standards.

**Figure 3:**
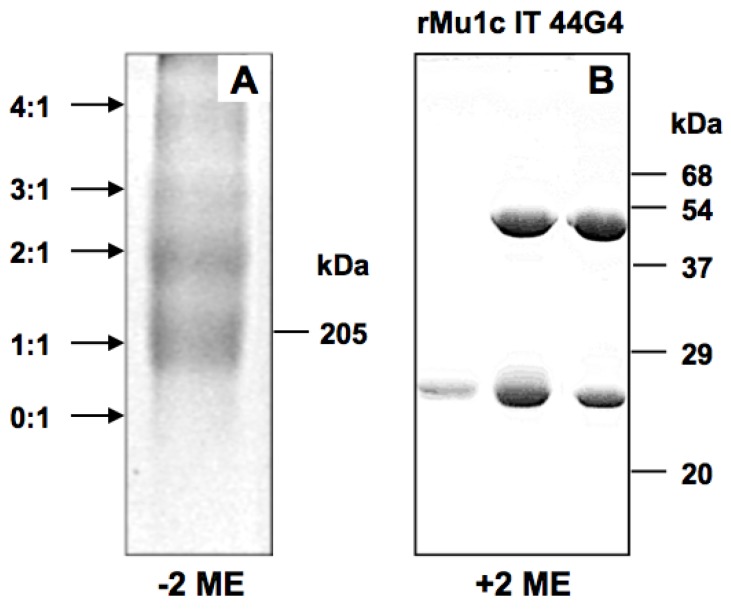
Analysis of rMU1-44G4 IT by SDS-polyacrylamide gel electrophoresis in the absence (**A**) or presence (**B**) of 2-mercaptoethanol (2-ME). (**A**) Electrophoresis in the absence of 2-ME was carried out with 7.5% (*w*/*v*) polyacrylamide gels; the rMU1/44G4 ratio is indicated on the left of the picture; (**B**): Electrophoresis in the presence of 2-ME was carried out with 18% (*w*/*v*) polyacrylamide gels. The numbers on the right of both gels indicated the apparent Mr of the markers used. The numbers on the left indicate the RIP/mAb stoichiometries.

Then 44G4 mAb was selected as anti-human CD105 since it is highly specific for human CD105 [22]. It was also derivatized with SPDP to introduce two to three sulfhydryl groups per molecule of 44G4 as reported for the other 44G4-containing immunotoxin ebulin-44G4 [21]. After the reaction between the activated rMU1 and the activated mAb, the reaction mixtures were subjected to chromatography through Sephacryl-S200 to isolate the reaction products. As shown in the upper panel of Figure 2, rMU1 was conjugated with 44G4, giving species that moved in the front as polymers. SDS-PAGE analysis at different acrylamide percentages revealed that peaks contained different chemical species from high molecular weight conjugates to isolated rMU1 and 44G4 (Figure 2, lower panel A). The size indicated in lower panel A of Figure 2 was estimated from the expected molecular weights for different stoichiometries between rMU1 and 44G4. Fractions collected as conjugates suitable for testing as cytotoxic species were those indicated by the C bar that were mixed and used as IT. D fractions were not used since they contained free 44G4 mAb which could compete with the immunotoxin for binding to CD105. As shown in Figure 3A, SDS-PAGE analysis of this mixture in the absence of 2-ME revealed conjugates with RIP/mAb stoichiometries of 1:1, 2:1, 3:1, 4:1. The IT used in the cell viability assays was a mixture of these species but essentially contained the 1:1 and 2:1 species. We further analyzed the components of the immunotoxin by SDS-PAGE in the presence of 2-ME to reduce disulphide linking. As shown in Figure 3B, electrophoresis revealed that reduction allowed the complete separation of both components of the IT rMU1 and 44G4. Conjugation of rMU1 with the mAb reduced its IC_50_ value on rabbit reticulocyte lysates from 14 ng/mL (approx. 5.2 × 10^−10^ M) to 24 ng/mL (approx. 9 × 10^−10^ M). Chemical conjugation of proteins with biological activity might affect and reduce and even eliminate their activities. In our case, the coupling of 44G4 to rMU1 did not significantly alter the translational inhibitory activity on translation by rabbit reticulocyte lysates. This indicated that, even after being linked to 44G4, the modified RIP molecules have free access to the substrate target loop of the 28S rRNA in the large ribosomal subunit.

**Figure 4:**
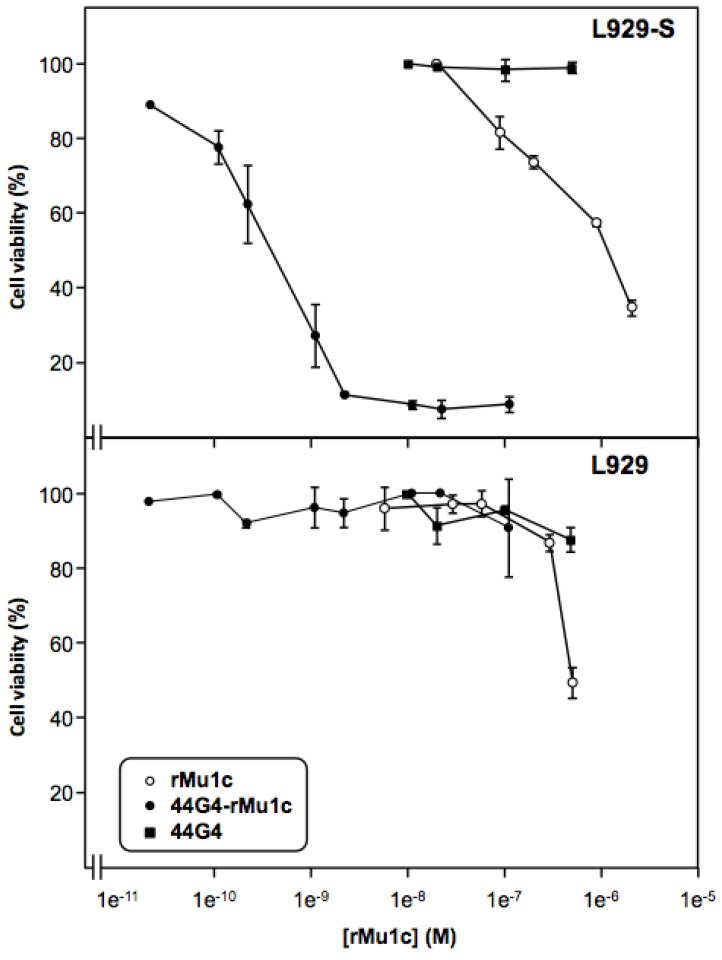
Cytotoxicity of the rMU1-44G4 immunotoxin on L929 expressing the short form of human endoglin (**upper panel**) and parental L929 mouse fibroblasts (**lower panel**). Growing cultures of transfected L929-hCD105+ and parental L929 cells were incubated for 3 h, either with rMU1, immunotoxin or 44G4 and the viability of cells was assayed with the WST-1 reagent as indicated in the Materials and Methods Section. Vertical bars indicate the ±SEM (*n* = 3).

Further, we assayed the cytotoxicity of the rMU1-44G4 IT on L929-shCD105+ mouse cells as a reduction of cell viability using the WST-1 reagent rather than inhibition of protein synthesis since viability is more sensitive to inhibitors than translation, most likely due to the pleiotropic cellular effects of RIPs [20,25]. As shown in Figure 4, rMU1-44G4 IT kills cells with an IC_50_ value of 3.9 × 10^−10^ M for L929-shCD105+ cells and >10^−7^ M for control untransfected L929 cells. Further, neither 44G4 mAb nor rMU1 alone display inhibitory action at concentrations such as those required to see full inhibition by the IT. The therapeutic window of IC_50_ between the CD105-expressing cells and the parental cells was over three orders of magnitude. These results point to the high specificity of the present IT for CD105.

The cytotoxicity of the present IT compares well with other ITs containing anti-CD105 antibodies such as those prepared with type 2 nigrin b or ebulin l as RIPs [12,20,21]. *In vivo* administration of anti-CD105 mAbs [13,26] and an anti-CD105-nigrin b IT [20] enabled murine tumors to be reduced and eliminated. CD105 has been proposed for targeted cancer treatment [11,13]. However, some concerns have arisen due to the presence of said marker in specialized cells, thus necessitating the need for further research. Kays and co-workers recently reported that CD105 is also a marker for human long-term repopulating hematopoietic stem cells [27]. Accordingly, some toxicity of anti-CD105 ITs on CD105-expressing human cells both *in vitro* and *in vivo* would be expected. In this line, previous results indicate that the IT 44G4-nigrin b is active on cultured L929-hCD105+ mouse fibroblast with an IC_50_ of 6 × 10^−10^ M and on human umbilical vein endothelial cells (HUVEC cells) with an IC_50_ of 6.4 × 10^−9^ M [20]. RIP delivery into the cell is thus mandatory for the inhibitory action of the IT, and such transport is fully mediated by the antibody binding to CD105 and internalization of the CD105-IT complex. This also suggests that the intracellular traffic of such complexes does not seem to be much affected by the type of RIP (type 2, type 1 or recombinant type 1 RIPs) linked to the antibody. However, since the precise mechanism of said complex pathway is not fully understood, further research is required.

Clinical trials have been conducted to test the efficiency of several immunotoxins *in vivo* in cancer patients, for instance in those suffering from leukemia and lymphoma [28,29,30]. Major obstacles encountered in the *in vivo* use of ITs lie in the appearance of vascular leak syndrome and the immunogenicity of IT components [31]. Attempts to circumvent vascular leak syndrome have been carried out, such as through chemical modifications of the RIP such as blocked ricin [32], the use of different RIPs, and the use of fusion proteins containing RIP domains [5,6]. In order to minimize neutralization, several strategies, such as reducing the size and humanization of the antibodies and preparing fusion proteins with reduced immunogenicity, have been used [33]. Moreover, research into the usefulness of anti-CD105 ITs will progress through technical advances in the delivery and efficiency testing of anti-CD105 drugs. In this line, developing a novel genetically engineered mouse model expressing humanized CD105 may enable more efficient therapy agents to be found [34]. Additionally, the present results open up the possibility of developing fusion proteins carrying the cytotoxic domain of rMU1 and binding domains specific for plasma membrane–inserted CD105.

In summary, cytotoxic anti-CD105 targeting therapy with an IT using a fully active recombinant type 1 RIP such as rMU1 increases the arsenal of tools for research into experimental cancer therapy. Further work will address the effects of the *in vivo* administration of the present IT to tumor-bearing mice, the pharmacokinetics and the immunogenic potential.

## 3. Materials and Methods

### 3.1. Chemicals, Reagents and Biological Materials

Common chemicals and biological materials were purchased from the same sources as reported elsewhere [12]. WST-1 reagent was from Roche Diagnostics, S.L. Life Science (Sant Cugat del Vallés, Spain) *N*-succinimidyl 3-(2-pyridyldithio)propionate was obtained from Sigma Chemical Co (St. Louis, MO, USA). Saporin S5 was a generous gift from Prof. F. Stirpe (Università di Bologna, Bologna, Italy). The hybridoma cell line 44G4 producing a monoclonal antibody (mAb) to hCD105 was kindly provided by Dr. Michelle Letarte through Dr. C. Bernabéu (Centro de Investigaciones Biologicas, CSIC, Madrid, Spain). It was grown as described elsewhere: Purification of 44G4 mAb was performed by protein A-Sepharose chromatography as described previously [21]. Mouse fibroblasts L929-shCD105+ expressing the short human form of CD105 were a generous gift from Dr. C. Bernabéu [35].

### 3.2. Recombinant Musarmin 1 (rMU1)

Highly purified rMU1 was prepared as reported previously [22]. Briefly, the strategy was as follows. The cloning vector containing the MU1 gene flanked by *NcoI* at the *N*-terminus and *Hind III* at the *C*-terminal sequence was amplified by PCR, ligated into pUC18 plasmid and sub-cloned. The plasmid containing MU1 gene was digested to release a restriction fragment of 830 bp which was then ligated to the expression plasmid pET-25b. The pET-25+MU1 plasmid was then introduced into the expression *Escherichia coli* strain BL21(DE3)pLysS. Expression was induced by isopropyl-β-d-thiogalactoside and the cells from the bacterial cultures were isolated, lysed by freeze-thawing and centrifuged to isolate the insoluble protein fraction containing the inclusion bodies. They were solubilized with cetyltrimethylammonium bromide and the insoluble material was removed by centrifugation. The resulting supernatant was dialyzed to remove the detergent and to allow refolding of rMU1. The protein was then purified from said supernatant by NaCl-gradient ion-exchange chromatography on SP-Sepharose followed by gel filtration through Superdex 75 GE Healthcare Europe GmbH (Barcelona, Spain). The yield was around 20 mg/L of bacterial culture of solubilized refolded rMU1.

### 3.3. Activation of the 44G4 Monoclonal Antibody

The 44G4 mouse monoclonal antibody (5 mg) was dissolved in 50 mM Na-borate (pH 9.0) buffer to obtain a concentration of 1.3 mg/mL. Five molar excess of the linker SPDP was added to the protein solution and the mixture was incubated for 1 h at 30 °C. Said solution was then dialyzed overnight at 4 °C against a 4 L of solution of 140 mM NaCl containing 5 mM sodium phosphate (pH 7.5). To determine the number of sulfhydryl groups per molecule, the derivatized protein was treated with 50 mM dithiothreitol to reduce the disulphide bridges and the released pyridine-2-thione was then determined at A_343_ (a control of absorption of pyridine-2-thione at A_280_ must be carried out), and compared to the derivatized protein. The ratio of linker bound to 44G4 was 1.9.

### 3.4. Preparation of Activated rMU1

rMU1 (15 mg) was dissolved in 50 mM Na-borate (pH 9.0) buffer to obtain a concentration of 4–5 mg/mL and activated with SPDP in the same way as for 44G4 monoclonal antibody and in a final volume of 1.5 mL. The activated rMU1 was dialyzed overnight at 4 °C against 4 L of 140 mM NaCl solution containing 5 mM sodium phosphate (pH 7.5). The ratio of the SPDP linker to the recombinant protein was determined as for 44G4 mAb, and was in the range of 1.0.

### 3.5. Preparation and Purification of rMU1-44G4 IT

First 3.7 mg of the activated 44G4 monoclonal antibody was reduced with 50 mM dithiothreitol for 30 min at room temperature to unblock the sulfhydryl groups. The protein was then chromatographed through a Sephadex G-25 column (bed volume 28 mL) GE Healthcare Europe GmbH (Barcelona, Spain) to remove the released low molecular weight material. That column previously equilibrated with a solution of 140 mM NaCl containing 5 mM sodium phosphate (pH 7.5) was eluted with the same solution. The protein was collected on 11 mg of the previously SPDP-activated and dialyzed rMU1. The conjugation reaction was carried out for 16 h with gentle stirring at room temperature. The solution was then concentrated to 7 mL with Amicon (YM10 membrane) Sigma Chemical Co (St. Louis, MO, USA), and centrifuged for 10 min at 13,000× *g* at 4 °C. The clear supernatant was chromatographed through a Superdex 200-HL column GE Healthcare Europe GmbH (Barcelona, Spain) equilibrated with 140 mM NaCl containing 5 mM sodium phosphate (pH 7.0), and eluted with the same buffer solution at 2 mL/min, collecting fractions of 5 mL. Fractions containing the immunotoxin were collected, pooled and further concentrated with Amicon YM10 up to 1 mL. The immunotoxin was then dialyzed, aliquoted, and stored at −80 °C. The yield of immunotoxin under our conditions was in the range of 1.2 mg.

### 3.6. Assay of Cell-Free Protein Synthesis

Cell-free protein synthesis was determined with a coupled transcription-translation *in vitro* assay using the rabbit reticulocyte lysate system as described elsewhere [36]. The reaction mixture contained in 8 μL was the following: 5 μL rabbit reticulocyte lysate, 6.5 U ribonuclease inhibitor, 4 U T7 RNA Polymerase, 0.25 μg Luciferase T7 plasmid, 0.4 mM rNTP’s each, 2 μM protein amino acids each, 10 mM Tris-HCl (pH 7.8), 0.2 mM spermidine, 28 mM KCl, 1 mM MgCl_2_ and nuclease-free water. The reaction mixtures were incubated at 30 °C for 10 min and chilled on ice. A volume of 2 μL of either water or dilutions of rMU1 of the rMU1-44G4 was added to the test tubes and samples were incubated at 30 °C for 30 min. A volume of 25 μL of water was added to the sample and 25 μL of the mixed dilution was removed and added to 25 μL of room temperature Luciferase Assay Reagent in a luminometer tube. The relative luciferase activities of the samples were determined in a Luminova 1254 luminometer (BIO ORBIT, Turku, Finland) for 10 s with an initial delay of 2 s. A volume of 2 μL of water was added to the reaction mixture to determine background luminescence.

### 3.7. Cytotoxicity of the rMU1-44G4 IT on L929-hCD105+ Cells

The cytotoxic activity of the immunotoxin, assessed as cell viability [19,20], was performed as follows. Briefly, 96-well plates were seeded with 3 × 10^3^ cells and 100 μL of culture medium per well. Culture medium for parental L929 cells was RPMI 1640 supplemented with 2 mM l-glutamine, 100 units/mL penicillin, 100 μg/mL streptomycin and 10% (*v*/*v*) heat-treated fetal calf serum. Transfected L929-CD105+ cells were incubated with the same culture medium as the parental ones but containing 400 μg/mL of geneticin. Incubation was for 24 h at 37 °C under 5% CO_2_. Wells were then washed with culture medium without fetal calf serum and then 100 μL of culture medium containing fetal calf serum was added and incubated with the corresponding amount of RIP or conjugate for 48 h at 37 °C under 5% CO_2_. At the end of incubation, 10 μL of WST-1 reagent (Roche Diagnostics, S.L. Life Science, Sant Cugat del Vallés, Spain) was added to each well and the plate was gently shacked for 2 min and then incubated for 2 h at 37 °C under 5% CO_2_. Reduction of the WST-1 reagent was quantified at 450 nm in an ELISA reader (Multiskan, Thermo Fisher Scientific, Alcobendas, Spain). Each experimental point was carried out in triplicate.

### 3.8. Other Procedures

Apparent Mr was determined by polyacrylamide gel electrophoresis in the presence of SDS (SDS-PAGE) and stained with Coomasie brilliant blue as described previously [31]. The electrophoresis Mr markers used were: myosin (apparent Mr 205,000); phosphofructoquinase (apparent Mr 116,000); bovine serum albumin (apparent Mr 68,000); l-glutamate dehydrogenase (apparent Mr 54,000); alcohol dehydrogenase (apparent Mr 37,000); carbonic anhydrase (apparent Mr 29,000); trypsin inhibitor (apparent Mr 20,100). Protein concentrations were determined by spectrophotometry [22].

## Abbreviations

The following abbreviations are used in this manuscript:

2-ME	2-mercaptoethanol
mAb	monoclonal antibody
RIP	ribosome inactivating protein
rMU1	recombinant musarmin 1
rNTP‘s	ribonucleotides
SDS-PAGE	sodium dodecyl sulfate polyacrylamide gel electrophoresis
SPDP	*N*-succinimidyl 3-(2-pyridyldithio)propionate
